# Antidepressant treatment and cultural differences - a survey of the attitudes of physicians and patients in Sweden and Turkey

**DOI:** 10.1186/1471-2296-11-93

**Published:** 2010-11-26

**Authors:** Alan G Wade, Paul CD Johnson, Alex McConnachie

**Affiliations:** 1 3 Todd Campus, West of Scotland Science Park, Glasgow G20 0KA, UK; 2 Robertson Centre for Biostatistics, Boyd Orr Building, University of Glasgow, Glasgow G12 8QQ, UK

## Abstract

**Background:**

The presenting symptoms of depression can be influenced by cultural differences. This study was conducted to compare the presenting symptoms and response to antidepressant medication of patients in Sweden and Turkey, two culturally different European countries.

**Methods:**

Recruitment was triggered when adult patients were diagnosed with a depressive or anxiety disorder by a primary care physician and prescribed an antidepressant. Physicians and patients recorded presenting symptoms and completed relevant questionnaires just before and 8 weeks after starting treatment with an antidepressant. These included the Hospital Anxiety and Depression Scale (HADS), the Clinical Global Impressions (CGI) scale, the Sheehan Disability Scale (SDS), and Likert scales gauging the importance of physical and psychological symptoms. Patients also rated severity of prominent symptoms (depression, anxiety, stress, sleep and pain) from zero to ten. The outcomes were compared between patients from Sweden and Turkey using Fisher's Exact test and two-sample t-tests.

**Results:**

The study was conducted in 460 patients (107, 23.3% in Sweden; 353, 76.7% in Turkey). Presenting symptoms differed between Sweden and Turkey, with Turkish patients more likely to present with physical symptoms, and report a higher number of physical symptoms (mean 2.4 vs. 1.4, p < 0.001). In both countries, the diagnosis made by the physician differed from that derived from the HADS score at the start of the study. The HADS diagnosis varied between the countries with significantly different proportions of patients in each country being diagnosed with depression alone, anxiety alone or depression with anxiety. While all symptoms improved after antidepressant treatment in both countries, Turkish patients showed a greater degree of response than Swedish patients in depression (p = 0.048), stress (p = 0.014) and pain (p < 0.001) as measured by the prominent symptoms assessment (PSA).

**Conclusions:**

The presenting symptoms of patients diagnosed with a depressive or anxiety disorder by a primary care physician and prescribed an antidepressant differ between Turkey and Sweden. Patients in Turkey were more likely to present with physical symptoms than patients in Sweden and present with more physical symptoms. After 8 weeks of antidepressant treatment, the improvement from baseline was greater in Turkish patients, and this was reflected in their improved functioning.

## Background

Depression is a common and disabling mental illness, which 'refers to a wide range of mental health problems characterised by the absence of a positive effect, low mood and a range of associated emotional, cognitive, physical and behavioural symptoms' [1]. Patients may present in many ways with physical, social or psychological symptoms to primary care physicians [2], and physical symptoms such as fatigue, headache, abdominal distress, or change in weight are often the presenting complaints in the primary care setting [3]. The diagnosis and classification of depression using DSM-IV and ICD-10 are based mainly on psychological symptoms [3,4], which may make it harder for primary care physicians to correctly diagnose depression in patients presenting with physical symptoms.

The symptomatic presentation of depression is known to vary between cultural groups [5,6]. People from non-Western cultures are more likely to use phrases that allude to physical sensations [6]. The World Organisation of Family Doctors (WONCA) culturally sensitive depression guideline notes that 'The primary care physician needs to understand the cultural, religious and gender paradigm that the individual brings to the consultation in order to increase the chance of establishing a therapeutic alliance that reduces the personal distance between physician and patient. This will maximise the chance of therapeutic success' [6].

Little is known about why a primary care physician prescribes an antidepressant in response to a diagnosis based on anxiety symptoms or depressive symptoms. Undoubtedly the decision is influenced by the interaction between physician and patient, the manner in which the patient presents and by differing cultural factors.

There are demographic and cultural differences between Sweden and Turkey. Sweden is a Western European country with a population with a median age of 41.5 years [7]. The majority of the population are Swedish born (87%) [8], live in urban areas (85%) and are Lutheran (87%) [7]. Turkey is an Eastern European country, which has a younger population with a median age of 27.7 years; with 31% living in rural areas [7]. The Turkish population are 70-75% Turkish ethnicity with 18% of Kurdish ethnicity and the population is almost entirely Muslim (99.8%).

The aim of the current study was to compare the following outcomes for patients prescribed antidepressants for 8 weeks in primary care in Sweden and Turkey:

1. Presenting symptoms of depression reported by the patient and their perceived relative importance to the patient, and patient-reported outcomes from the Hospital Anxiety and Depression Scale (HADS), Sheehan Disability Scale and a prominent symptoms assessment scale

2. Presenting symptoms of depression noted by the patient's physician and their perceived relative importance

3. Presumptive diagnosis by the physician compared with a diagnosis determined by the HADS scale

4. Changes in symptoms after 8 weeks of antidepressant treatment as assessed by patient-reported outcomes and the patient's physician

## Methods

### Study design

This was an 8-week naturalistic study designed to compare presenting symptoms and response to antidepressant medication in adult patients prescribed an antidepressant by their primary care physician in Sweden and Turkey, two culturally different European countries. The study was approved by the appropriate ethical committees in Sweden (Uppsala Regional Ethics Committee) and Turkey (Eftal Training and Research Hospital, Istanbul) in accordance with the International Conference on Harmonization (ICH) guidelines for Good Clinical Practice (GCP).

### Patients

Recruitment to the study was triggered by primary care physicians first prescribing an antidepressant to patients aged 18-80. There were no specific exclusion criteria. Concomitant medications, including anxiolytics and analgesics, were allowed. A total of 460 consecutive consenting patients, 107 of whom (23.3%) were recruited in Sweden and 353 (76.7%) in Turkey, were enrolled in the study by 65 primary care physicians (43 in Turkey and 22 in Sweden) between October 2004 and August 2006.

### Ethics

The study was approved by the appropriate ethical committees in Sweden and Turkey in accordance with the International Conference on Harmonization (ICH) guidelines for Good Clinical Practice (GCP) [9].

### Outcome measures

Following informed patient consent, parallel questionnaires were independently completed by the physician and patient. The diagnosis triggering the decision to prescribe an antidepressant was recorded by the physician, and for comparison, the patient completed the self-rating Hospital Anxiety and Depression Scale (HADS) [10]. Depression without anxiety was defined as a HADS-Depression score ≥11 and a HADS-Anxiety score <11; depression with anxiety was defined as a score ≥11 for both the HADS-Depression and HADS-Anxiety subscales; and anxiety without depression was defined as a HADS-Anxiety score ≥11 and a HADS-Depression score <11. Patients scoring <11 on both scales were 'unassigned'. The degree of illness was assessed by the physician using the Clinical Global Impression Severity (CGI-S) scale [11] and by patients completing a battery of five scales of prominent symptoms (depression, anxiety, stress, sleep and pain, rated from zero to ten). In a separate analysis we have shown that the prominent symptoms assessment scale reliably reflects prominent symptoms of depression (unpublished data). The physical and psychological symptoms reported by the patient on presentation were also recorded by the physician. The importance of a range of psychological (depression, anxiety, stress and sleep problems) and physical (fatigue, gastro-intestinal [GI] disturbance, headache, pain and backache) symptoms was assessed by both the physician and the patient on a scale from 1-5. The Sheehan Disability Scale (SDS) was used as a self-rated scale to assess the degree of disability in relation to work, social life and family life, based on the previous week [12]. Each of these 3 domains was rated from 0 to 10 (no impairment to most severe impairment). The same questionnaires were also completed after 8 weeks to provide information about patient outcome and relative changes in patient symptoms. In addition, patients self-rated their overall improvement using the CGI improvement scale (CGI-I) at 8 weeks, and physicians assessed compliance with antidepressant medication (defined as the patient reporting that they were still taking antidepressant medication at Week 8).

### Statistical methods

Differences between countries were explored initially using Fisher's Exact test and two-sample t-tests. Significant associations were investigated further using linear mixed effects regression models fitted using restricted maximum likelihood, adjusting for the effects of age and sex, and for heterogeneity between physician practices.

## Results

### Patient population

The demographic characteristics of the study population at baseline are summarised in Table 1. The proportion of women recruited in Turkey was higher than in Sweden, although most patients in both countries were women. While the majority of patients in both countries were aged between 35-49 years, the mean age of patients recruited in Turkey was 11 years lower than in Sweden.

**Table 1:** Demographic and mental health characteristics of the study population on prescription of an antidepressant (baseline), by country

Characteristic		Sweden (n = 107)	Turkey (n = 353)	P-value
Sex, n (%)	Female	58 (55.2%)	269 (76.4%)	<0.001

Age	Mean ± SD (n)	51.1 ± 14.7 (100)	39.9 ± 11.5 (340)	<0.001

CGI-Severity ^a^	Mean ± SD (n)	4.4 ± 0.8 (105)	5.1 ± 1.1 (344)	<0.001

Medications prescribed, n (%)	SSRIs	66 (88.0%)	275 (93.2%)	0.149
	TCAs	0 (0.0%)	5 (1.7%)	0.588
	Venlafaxine	3 (4.0%)	6 (2.0%)	0.395
	Mirtazapine	7 (9.3%)	8 (2.7%)	0.017
	Other ADs	0 (0.0%)	20 (6.8%)	0.018
	Anxiolytics	5 (6.7%)	52 (17.6%)	0.019
	Hypnotics	11 (14.7%)	2 (0.7%)	<0.001
	Analgesics	10 (13.3%)	15 (5.1%)	0.018

Presenting symptoms (psychological), n (%) ^a^	Depression	70 (65.4%)	267 (75.6%)	0.046
	Anxiety	55 (51.4%)	188 (53.3%)	0.742
	Sleep	55 (51.4%)	201 (56.9%)	0.320

Presenting symptoms (physical), n (%) ^a^	Fatigue	47 (43.9%)	200 (56.7%)	0.027
	Pain	40 (37.4%)	209 (59.2%)	<0.001
	Gastro-intestinal	21 (19.6%)	150 (42.5%)	<0.001
	Cardiovascular	8 (7.5%)	86 (24.4%)	<0.001
	CNS	29 (27.1%)	194 (55.0%)	<0.001

Number of psychological symptoms ^a^	Mean ± SD (n)	1.7 ± 0.9 (107)	1.9 ± 0.9 (353)	0.067

Number of physical symptoms ^a^	Mean ± SD (n)	1.4 ± 1.1 (107)	2.4 ± 1.3 (353)	<0.001

HADS-Depression ^b^	Mean ± SD (n)	10.3 ± 4.2 (99)	12.8 ± 4.5 (335)	<0.001

HADS-Anxiety ^b^	Mean ± SD (n)	12.9 ± 4.0 (99)	12.6 ± 4.0 (335)	0.577

Prominent symptoms-Depression ^b^	Mean ± SD (n)	5.9 ± 2.2 (97)	6.8 ± 2.6 (342)	0.005

Prominent symptoms-Anxiety ^b^	Mean ± SD (n)	5.7 ± 2.4 (101)	6.1 ± 2.8 (339)	0.259

Prominent symptoms-Stress ^b^	Mean ± SD (n)	6.2 ± 2.6 (101)	6.9 ± 2.5 (341)	0.010

Prominent symptoms-Sleep ^b^	Mean ± SD (n)	5.9 ± 2.9 (101)	6.4 ± 2.9 (341)	0.158

Prominent symptoms-Pain ^b^	Mean ± SD (n)	4.8 ± 3.1 (101)	6.2 ± 2.8 (342)	<0.001

SDS-Work ^b^	Mean ± SD (n)	6.0 ± 3.2 (98)	6.0 ± 2.7 (332)	0.873

SDS-Social ^b^	Mean ± SD (n)	6.9 ± 2.5 (102)	6.8 ± 2.5 (342)	0.564

SDS-Family ^b^	Mean ± SD (n)	6.6 ± 2.5 (102)	6.5 ± 2.6 (342)	0.882

^a ^As assessed/recorded by the primary care physician

^b ^As assessed/recorded by the patient

Abbreviations: AD: antidepressant, CGI-S: Clinical Global Impression Severity Scale, HADS: Hospital Anxiety and Depression Scale, SD: standard deviation, SDS: Sheehan Disability Scale, SSRI: selective serotonin reuptake inhibitor, TCA: tricyclic antidepressant

P-values from two sample t-test

### Presenting symptoms

A higher proportion of Turkish patients (75.6%) than Swedish patients (65.4%) presented with symptoms of depression (p = 0.046). There were no other significant differences between countries with respect to the category or number of presenting psychological symptoms at baseline (Table 1).

**Figure 1:**
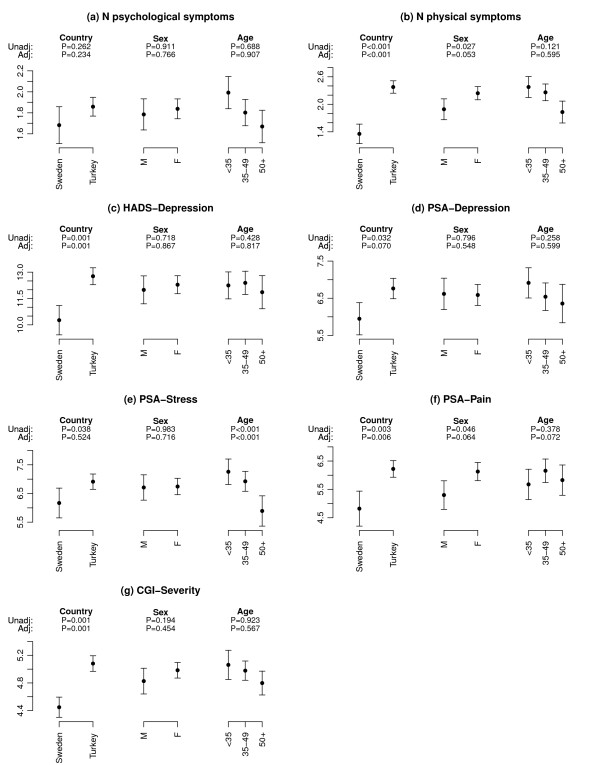
**Mean (95% CI) outcome variables at presentation, by country, and by sex and age category within each country**. The number of psychological and physical symptoms reported at presentation; the HADS depression score and prominent symptoms scores for depression, stress and pain score assessed by the patient; and the CGI-severity (CGI-S) score assessed by the physician are shown. P-values are from tests of association between outcome and the covariates country, sex and age from three univariate linear mixed effects regression models (unadj), and from a multivariate model where the effect of each covariate is adjusted for the effects of the other two (adj).

Turkish patients were more likely to present with physical symptoms of fatigue, pain, GI disturbance, cardiovascular and CNS problems than Swedish patients (Table 1). The mean number of physical symptoms reported by patients in Turkey was also greater than in Sweden (2.4 vs. 1.4, p < 0.001; Figure 1b, Table 1).

The duration of the presenting symptoms prior to a patient first visiting the physician did not differ significantly between countries; the majority of patients (84.1%) had experienced symptoms for more than one month.

Patients in Turkey scored significantly higher than their Swedish counterparts on the depression, stress and pain components of the patient-reported prominent symptoms (Figure 1d-f, Table 1), with no evidence of association between the prominent symptoms depression scores and age or sex.

The proportion of patients with severe or extreme illness as assessed by the physician using the CGI-S was significantly greater in Turkey (36.6%) than in Sweden (4.8%), (p < 0.001), as was the mean CGI-S score (Figure 1g, Table 1).

### Importance of symptoms

**Figure 2:**
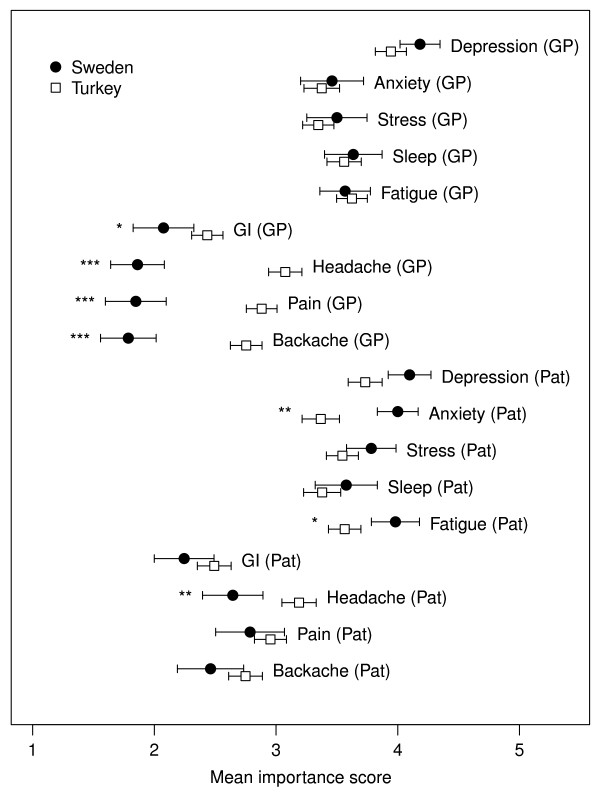
**Mean (95% CI) importance of symptoms in Swedish and Turkish patients, assessed by the physician (GP) and the patient (Pat)**. Asterisks indicate a significant difference between countries (mixed effects linear regression model adjusted for age and sex: *p < 0.05; **p < 0.01; ***p < 0.001). Importance was rated on a 5 point scale (1, not present; 2, present but not important; 3, present; 4, important but not major complaint; 5, very important). GI: gastro-intestinal symptoms.

The importance of physical symptoms was rated higher in Turkey than in Sweden (mean values of 2.98 vs. 2.42, p < 0.001) by the physician, while the patient's rating did not differ between countries. A contrasting pattern was observed for psychological symptoms. There was no difference between countries in the physician's mean assessment of importance, but patients rated the importance of psychological symptoms higher in Sweden than in Turkey (mean values of 3.87 vs. 3.49, p < 0.001). The general pattern of physicians rating the importance of physical symptoms higher in Turkey and patients rating psychological symptoms as more important in Sweden reflects the between-country differences apparent in the individual symptoms (Figure 2). An exception to this pattern was fatigue, which follows the pattern of psychological symptoms, and was rated as more important by patients in Sweden than in Turkey.

### Diagnosis (Physician and HADS)

There was no significant difference between the countries in the physician's initial diagnosis. However, in both countries the physician's diagnosis differed from the diagnosis obtained by the HADS, with anxiety being under-diagnosed by physicians (Table 2). A statistically significant difference between the countries in diagnosis as measured by the HADS at the first visit was observed (p < 0.001). Figure 1c shows that patients in Turkey scored significantly higher on the HADS-Depression scale than patients in Sweden (see also Table 1). No associations were found between HADS-Depression scores and age or sex.

**Table 2:** Number (%) of patients with main diagnosis according to the primary care physician and the HADS categories, and agreement between the physician and HADS

Diagnostic method		Sweden (n = 107)	Turkey (n = 353)	P-value
Physician diagnosis, n (%)	Depression alone	53 (52.0%)	155 (45.2%)	
	Depression + anxiety	46 (45.1%)	158 (46.1%)	0.104
	Anxiety alone	3 (2.9%)	30 (8.7%)	

HADS diagnosis, n (%)	Depression alone	4 (4.0%)	56 (16.7%)	
	Depression + anxiety	43 (43.4%)	176 (52.5%)	<0.001
	Anxiety alone	33 (33.3%)	65 (19.4%)	
	Non-assigned	19 (19.2%)	38 (11.3%)	

Physician-HADS agreement, n (%)	Same diagnosis	33 (35.1%)	162 (49.8%)	0.014

Abbreviations: HADS: Hospital Anxiety and Depression Scale

P-values from tests of equal prevalence (Fisher Exact test)

### Medication

Selective serotonin reuptake inhibitors (SSRIs) were the most commonly prescribed class of antidepressant at the first visit, with the majority of patients in both Sweden (88.0%) and Turkey (93.2%) being prescribed at least one SSRI (Table 1). Antidepressants other than SSRIs, venlafaxine or mirtazapine were not prescribed in Sweden, whereas tricyclic antidepressants (TCAs) and other antidepressants were prescribed for a small minority of Turkish patients.

### Changes in symptoms after 8 weeks

The proportion of patients returning for their 8-week visit was higher in Turkey than in Sweden (92.9% vs. 76.6%, p < 0.001). We investigated the potential for the higher dropout rate in Sweden to bias the results by comparing the baseline characteristics listed in Table 1 between returners and non-returners within each country (data non shown). In Sweden, none of the outcomes differed significantly, while in Turkey non-returners had lower CGI severity, were less likely to have presented with pain and had fewer physical presenting symptoms. Substantial bias is therefore unlikely because of the low dropout rate in Turkey and the similarity between returners and non-returners in Sweden.

The results of the overall improvement in the patient's condition using the CGI-I scale, as assessed by the physician and the patient are shown in Table 3. The distributions of the assessments (very much improved, much improved, minimal improvement or same/worse), were similar between physicians and patients, and in agreement 70.3% of the time; physician-patient agreement was higher in Turkey than in Sweden (73% vs. 61%, p = 0.040).

**Table 3:** Number (%) of patients with CGI-I rating after 8 weeks of antidepressant treatment according to the primary care physician and the patient, and agreement between physician and patient

Measure		Sweden (2)	Turkey (n = 328)	P-value
Physician CGI-I, n (%)	Very much improved	10 (12.2%)	73 (23.1%)	
	Much improved	40 (48.8%)	184 (58.2%)	0.001
	Minimal improved	23 (28.0%)	46 (14.6%)	
	Same/worse	9 (11.0%)	13 (4.1%)	

Patient CGI-I, n (%)	Very much improved	7 (8.9%)	85 (27.2%)	
	Much improved	31 (39.2%)	173 (55.3%)	<0.001
	Minimal improved	31 (39.2%)	27 (8.6%)	
	Same/worse	10 (12.7%)	28 (8.9%)	

Physician-patient agreement, n (%)	Same assessment	48 (60.8%)	227 (72.8%)	0.040

P-values from tests of equal prevalence (Fisher Exact test)

**Figure 3:**
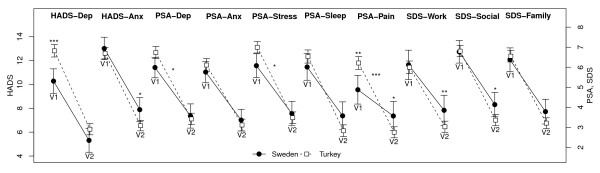
**Mean (±95% CI) HADS, prominent symptoms and SDS scores in Swedish and Turkish patients**. Asterisks above points indicate a significant difference between countries in mean score at Visit 1 (baseline) and Visit 2 (Week 8), and in mean change between visits. Asterisks between points indicate significant differences in the degree of change of symptoms between countries. (mixed effects linear regression model adjusted for age and sex: *p < 0.05; **p < 0.01; ***p < 0.001).

Comparison of the patients' mean rating of the importance of symptoms at the first and second visits showed a reduction in all symptoms from baseline (data not shown). The changes in mean HADS, prominent symptoms and SDS scores from baseline to Week 8 are compared by country in Figure 3. The degree of improvement was greater in Turkish patients than in Swedish patients across all measures, although this was significant only for three prominent symptoms (PSA-depression: p = 0.048; PSA-stress: p = 0.014; PSA-pain: p < 0.001). Having had higher HADS-Depression scores at the first visit, Turkish patients improved to near parity with Swedish patients at Week 8 (Figure 3). However, the degree of improvement did not differ significantly between Turkey and Sweden (mean 6.57 vs. 4.97, p = 0.076). There were no significant differences between the countries in mean SDS scores at baseline, but at Week 8 patients in Turkey had lower mean scores on the Work (p = 0.009) and Social (p = 0.039).

### Medication at Week 8

Over 90% of patients returning for the second visit at Week 8 received a further prescription of antidepressant medication. Table 4 compares the use of concomitant medication at baseline and Week 8. Approximately half the patients initially prescribed anxiolytics received a further prescription (3.6% in Sweden and 7.9% in Turkey). In addition, 10.7% of Swedish patients received a further prescription for hypnotics and 12.5% for additional analgesics. Repeat prescription of hypnotics and analgesics was minimal in Turkey.

**Table 4:** Number (%) of patients with concomitant medication of anxiolytics, hypnotics and analgesics on prescription of an antidepressant (baseline) and after 8 weeks of antidepressant treatment

Concomitant medication	Baseline			Week 8		
	
	Sweden n (%)	Turkey n (%)	p-value	Sweden n (%)	Turkey n (%)	p-value
Anxiolytics	5 (6.7%)	52 (17.6%)	0.019	2 (3.6%)	22 (7.9%)	0.394
Hypnotics	11 (14.7%)	2 (0.7%)	<0.001	6 (10.7%)	0 (0.0%)	<0.001
Analgesics	10 (13.3%)	15 (5.1%)	0.018	7 (12.5%)	5 (1.8%)	0.001

Treatment compliance (patient's reported adherence to the prescribed course of antidepressant treatment assessed at the second visit at Week 8) was high overall but did not differ significantly between Turkey and Sweden (93.7% vs. 90.2%, p < 0.329).

## Discussion

This study showed no significant difference between primary care physicians in Turkey and Sweden in assigning a diagnosis of depression, anxiety or mixed depression/anxiety to patients prescribed an antidepressant. The patient, however, reported that presenting symptoms, HADS diagnosis and treatment response differed between the two countries. Patients recruited in Turkey were significantly more likely than Swedish patients to present with physical symptoms. They also reported a higher number of physical symptoms and perceived them to be more important than their Swedish counterparts.

The differences noted between the study participants recruited in Sweden and Turkey may reflect the relative demography of the two countries' populations, although cultural differences in the other factors leading to prescription of an antidepressant - such as the incidence of depression, tendency to visit a physician and accuracy of diagnosis - could also have contributed to this pattern.

Based on the self-reported HADS scores, using the high score of 11 as indicating caseness [13], significantly different proportions of patients in each country were prescribed antidepressants for depression alone, anxiety alone or depression with anxiety. Physicians in both countries were more likely to make a diagnosis of depression whilst acknowledging the presence of anxiety symptoms and apparently recognising their importance. This tendency may reflect the increasing recommendation for and use of antidepressants for symptoms of anxiety.

Turkish patients also tended to show a greater degree of response after 8 weeks of antidepressant treatment than Swedish patients and one might speculate that this could be associated with the higher proportion of patients in the Swedish cohort self-reporting anxiety symptoms which may be less responsive to antidepressant treatment. Whatever the reason, the symptomatic improvement observed in Turkish patients is reflected in significantly better functioning at Week 8 within the work and social domains of the SDS in comparison to the Swedish population (Figure 3).

Somatisation was more frequent among Turkish patients than among Swedish patients and this is a common explanation for the apparent under-recognition of depressive disorders in non-Western cultures [6]. Patients and physicians in Turkey - but not in Sweden - often agreed upon the importance assigned to pain, suggesting that Turkish physicians are cognizant of the patient's cultural background and associated tendency to allude to physical rather than psychological symptoms.

Analysis of the HADS and prominent symptoms results reveals that symptoms of depression were more severe at baseline in Turkey compared with Sweden. Furthermore, the extent improvement in depression symptoms after prescription of an antidepressant for 8 weeks was significantly greater in Turkish patients than in Swedish patients. This suggests that Turkish patients responded better to treatment, or at least were more satisfied with the treatment, or gave more extreme ratings of symptoms when depressed or anxious. It is possible that these results do not indicate that Turkish patients are more depressed at baseline but that the HADS does not translate well in this culture. However, this is not supported by the prominent symptoms result, which showed a marginally non-significant difference, or by the CGI-S. The more likely explanation is that Turkish patients are more depressed because they go to their physician less readily or because Turkish primary care physicians have a higher prescribing threshold. Nevertheless Ozmen et al. concluded that the general public in Turkey believe that depression is a treatable illness [14]. It is, therefore, possible that these results may in part be due to this cultural belief, although Ozmen suggests that the public has an optimistic view about the prognosis and treatment of depression, whatever their cultural characteristics.

A further possibility is that differences in the choice of prescribed medication influence these results. The majority of physicians in both countries prescribed SSRIs, but some significant differences were noted between the countries in other drugs prescribed. For example, anxiolytics were more commonly co-prescribed in Turkey, while hypnotics and analgesics were more common in Sweden, although there was no difference between countries in the prevalence of anxiety or sleep as presenting complaints, nor in their severity as measured by the HADS and prominent symptoms scale. Pain was a more common presenting complaint in Turkey compared with Sweden, which seems to contradict the lower level of analgesics prescribed, unless these drugs were already prescribed or purchased over the counter and having the desired effect in Swedish patients.

The difference in presenting symptoms observed in this study most likely reflect cultural differences between Sweden and Turkey. In countries populated by diverse ethnic groups, it is important that physicians are aware of cultural differences in patients' presentation and expectations. The disparity observed between the diagnosis recorded by physicians and that derived from the HADS scale may have implications for both clinical practice and multicenter clinical studies in depression and anxiety conducted across countries with differing cultural backgrounds or across differing ethnic groups within a country.

### Limitations

The practices chosen were self selected and cannot be guaranteed to be representative of the totality of the primary care provision in either country.

This was a naturalistic study where the diagnosis of anxiety or depression was not substantiated by formal rating scales.

The report of adherence to medication is based solely on the patient report which has been shown to be potentially unreliable.

## Conclusions

This study shows that the presenting symptoms of patients diagnosed with a depressive or anxiety disorder by a primary care physician and prescribed an antidepressant differ between Turkey and Sweden. Patients in Turkey were more likely to present with physical symptoms than patients in Sweden and presented with more physical symptoms. After 8 weeks of antidepressant treatment, the improvement from baseline was greater in Turkish patients, and this was reflected in their improved functioning.

## Competing interests

The authors declare that they have no competing interests.

## Authors' contributions

AGW designed the study. PCDJ and AM were responsible for the statistical analyses. AGW prepared the initial draft of the manuscript with Dr Susan Downie; PCDJ and AM read and commented upon the draft of the manuscript. All authors have read and approved the final manuscript.

## Pre-publication history

The pre-publication history for this paper can be accessed here:

http://www.biomedcentral.com/1471-2296/11/93/prepub
